# Targeting and Cytotoxicity of SapC-DOPS Nanovesicles in Pancreatic Cancer

**DOI:** 10.1371/journal.pone.0075507

**Published:** 2013-10-04

**Authors:** Zhengtao Chu, Shadi Abu-Baker, Mary B. Palascak, Syed A. Ahmad, Robert S. Franco, Xiaoyang Qi

**Affiliations:** 1 Division of Hematology/Oncology, Department of Internal Medicine, University of Cincinnati College of Medicine, Cincinnati, Ohio, United States of America; 2 Department of Surgery, University of Cincinnati College of Medicine, Cincinnati, Ohio, United States of America; 3 Division of Human Genetics, Department of Pediatrics, Cincinnati Children’s Hospital Medical Center, University of Cincinnati College of Medicine, Cincinnati, Ohio, United States of America; Wayne State University School of Medicine, United States of America

## Abstract

Only a small number of promising drugs target pancreatic cancer, which is the fourth leading cause of cancer deaths with a 5-year survival of less than 5%. Our goal is to develop a new biotherapeutic agent in which a lysosomal protein (saposin C, SapC) and a phospholipid (dioleoylphosphatidylserine, DOPS) are assembled into nanovesicles (SapC-DOPS) for treating pancreatic cancer. A distinguishing feature of SapC-DOPS nanovesicles is their high affinity for phosphatidylserine (PS) rich microdomains, which are abnormally exposed on the membrane surface of human pancreatic tumor cells. To evaluate the role of external cell PS, *in vitro* assays were used to correlate PS exposure and the cytotoxic effect of SapC-DOPS in human tumor and nontumorigenic pancreatic cells. Next, pancreatic tumor xenografts (orthotopic and subcutaneous models) were used for tumor targeting and therapeutic efficacy studies with systemic SapC-DOPS treatment. We observed that the nanovesicles selectively killed human pancreatic cancer cells *in vitro* by inducing apoptotic death, whereas untransformed cells remained unaffected. This *in vitro* cytotoxic effect correlated to the surface exposure level of PS on the tumor cells. Using xenografts, animals treated with SapC-DOPS showed clear survival benefits and their tumors shrank or disappeared. Furthermore, using a double-tracking method in live mice, we showed that the nanovesicles were specifically targeted to orthotopically-implanted, bioluminescent pancreatic tumors. These data suggest that the acidic phospholipid PS is a biomarker for pancreatic cancer that can be effectively targeted for therapy utilizing cancer-selective SapC-DOPS nanovesicles. This study provides convincing evidence in support of developing a new therapeutic approach to pancreatic cancer.

## Introduction

Pancreatic cancer is the fourth leading cause of cancer deaths, with a 5-year survival of less than 5% [1], [2], [3]. It is usually asymptomatic in the early stages, while frequently invading regional lymph nodes and liver, and less often the lungs and visceral organs. Current multi-modal strategies, including surgery, chemotherapy, and radiation therapy, have failed to improve long-term survival. The current standard of treatment, the nucleoside analog gemcitabine [4], prolongs survival by only several months. Despite exhaustive efforts to map the genetic alterations associated with pancreatic cancer growth, few promising drug targets have been reported, and new, effective treatments are urgently needed. Experimental therapeutic strategies include small and large molecule inhibitors of oncogenic pathways, anti-angiogenic agents, vaccination/immunotherapy, gene therapies, and many others, but no clearly superior therapies have emerged.

In the last two decades, cellular membranes have become targets for anti-cancer drugs. Several lines of evidence have suggested a linkage between cellular membrane abnormalities and ceramide-mediated induction of apoptosis in tumors [5], [6], [7], [8], [9]. Based on these observations, agents that interfere with cellular membranes have been developed to modulate membrane organization, fluidity, metabolism, and signal transduction [10], [11], [12]. Little is known, however, of the underlying signaling pathways impacted by membrane-targeted anti-neoplastic agents.

We have been working to develop a novel biotherapeutic drug that can selectively target the cell membrane of pancreatic tumors and effectively destroy malignant pancreatic cells without harming normal tissues and cells. This agent is composed of two purified natural cellular components – a small natural protein (saposin C, SapC) and a natural lipid (dioleoylphosphatidylserine, DOPS) – which we assemble into cancer-selective nanovesicles (SapC-DOPS). SapC is a small nonenzymatic glycoprotein present in all normal tissues that acts as a biological activator of lysosomal enzymes [13]. The functional organization of SapC includes a membrane fusogenic domain and a region for activation of lysosomal enzymes [14], [15]. The *N*-linked glycosylation is not essential for SapC activity [16], [17]. SapC enhances degradation of glucosylceramide, sphingomyelin, and galactosylceramide to ceramide via acid β-glucosidase, acid sphingomyelinase, and acid β-galacotsylceramidase, respectively [13], [18], [19]. In patients with lysosomal storage diseases, SapC accumulates along with glycosphingolipids in macrophages [20] and it appears that excessive SapC and lipids may be toxic for these cells.

A general property of saposins is their lipid membrane binding activity [21] since they play important roles in lipid transport [22], lipid microdomain assembly [23], and reorganization of biological membranes [24], [25], [26]. SapC preferentially interacts with unsaturated, negatively charged phospholipids (such as DOPS), at acidic pH [15], [21]. This interaction is required for SapC activation of lysosomal enzymes.

We hypothesized that because phosphatidylserine (PS) is relatively abundant on the surface of cancer cells and tissues [27], [28], it would provide a tumor-specific target for SapC. In comparison to untransformed cells, neoplastic cells are hypermetabolic and thus produce significant amounts of acid and carbon dioxide (CO_2_) as by-products of anaerobic glycolysis and aerobic respiration, respectively. Hydrogen ions accumulate in tumor tissues and as a result, the mean extracellular pH in solid tumors is lower (pH ∼ 6) than that in normal tissues (pH ∼ 7) [29], [30]. Cancerous cells also display other unique properties, such as generalized membrane alterations [31], [32], [33] and “leakiness” of lysosomal enzymes [31]. Intriguingly, several lysosomal hydrolases are elevated in tumor tissues [34], [35]. Therefore, a unique acidic microenvironment with extracellular leakage of lysosomal enzymes makes tumor tissue an optimal target for SapC [9].

In a previous study, we found that SapC-DOPS induced apoptosis in multiple cancer cell types, including neuroblastoma, malignant peripheral nerve sheath tumor, and breast cancer cells, while sparing normal cells and tissues [36]. The mechanism of SapC-DOPS induction of apoptosis was determined to be, in part, through elevation of intracellular ceramides, followed by caspase activation. We also investigated the antitumor efficacy and systemic biodistribution of SapC-DOPS nanovesicles in preclinical cancer models [9], [36], [37]. We found that SapC-DOPS nanovesicles significantly targeted and inhibited the growth of preclinical xenografts of neuroblastoma, malignant peripheral nerve sheath tumor, and skin cancer and showed no toxic effects in nontumor tissues [9], [36], [37].

In the current study, we evaluated the anti-cancer utility of SapC-DOPS, and its *in vitro* and *in vivo* cancer-targeting and anti-neoplastic activity against human pancreatic cancer cells and pancreatic tumor xenografts.

## Materials and Methods

### Cell Cultures

Human pancreatic cell lines (MiaPaCa-2, PANC-1, BxPC-3, Capan-1, AsPC-1, HPAF-II, Hs766T) from American Type Culture Collection (ATCC, Manassa, VA) were cultured with Dulbecco’s Modified Eagle Medium (DMEM) supplemented with 10% fetal bovine serum, 100 units of penicillin/ml, and 10 mg of streptomycin/ml. Human pancreatic ductal epithelium (HPDE) was kindly provided by A. Lowy (Moores UCSD Cancer Center, La Jolla, CA), and grown as described in the literature [38]. Human pancreatic cfPac1-Luc3 cell line was kindly provided by O. Wildner (Ruhr-University Bochum, Bochum, Germany) [39]. All cells were cultured at 37°C in 5% CO_2_. No cross-contamination was found in these cells.

### Preparation of Proteins and Nanovesicles

SapC was produced as previously described [17] with modifications. Briefly, recombinant saposins were expressed using the pET system in *E. Coli* cells. Expressed proteins were purified on a nickel column and completely desalted with C4 reverse-phase high performance liquid chromatography (HPLC). After lyophilization, saposin powder was used and its concentration was determined by its weight. All phospholipids were purchased from Avanti Polar Lipids (Alabaster, AL). Bath sonication was used to form SapC-DOPS nanovesicles as previously described with minor modifications [40]. After solvent removal under nitrogen gas, phospholipids were mixed with pure saposin proteins in 20 µl of acid buffer (pH 5) and quickly diluted in 50X volume of physiological aqueous solution. The protein-lipid mixture was then gently sonicated and the two components readily assembled into nanovesicles. The sonicated nanovesicles can be used after storing at 4°C for at least a week. For long-term storage, stable lyophilized SapC-DOPS samples were prepared with a water-organic cosolvent system. After removing the solvent, dried DOPS was dissolved in 80% tert-butyl alcohol. SapC and sucrose (10 mg/ml) solution were made in water. DOPS and SapC/sucrose (vol:vol = 1∶0.6) mixture was lyophilized to a powder cake in a freeze dryer (VirTis Unitop, The VIRTIS Co., Gardiner, NY). For animal injection, the cake was rehydrated in phosphate buffered saline (PBS) to form SapC-DOPS nanovesicles. Those nanovesicles showed the same tumor-targeting and cytotoxicity *in vitro* and *in vivo*.

SapC-DOPS nanovesicles were characterized and monitored by an N4 plus particle size analyzer as previous described [9], [36]. Stable SapC-DOPS nanovesicles have an average diameter of approximately 200 nm with a mean zeta potential at −42. SapC binds the lipid molecules and spontaneously incorporates into the lipid bilayer of the liposomes upon sonication. Following sonication and ultracentrifugation to pellet SapC-DOPS coupled liposomes, no SapC was detected in the supernatant fraction, implicating a very high loading/coupling efficiency [9]. Dry SapC was dissolved in PBS solution for SapC treatment without DOPS. DOPS without SapC was prepared using the same preparation procedure for SapC-DOPS nanovesicles.

### Live Imaging

#### Fluorescent labeling of SapC-DOPS nanovesicles

In some portions of the experiment, the SapC-DOPS nanovesicles were fluorescently labeled with a stable and high intensity dye, CellVue® Maroon (CVM, Molecular Targeting Technology Inc., Exton, PA - Excitation max = 647 nm; Emission max = 677 nm [9], [36]). CVM has demonstrated utility in imaging subcutaneous tumors, as well as hidden, orthotopically implanted, and metastatic tumors. The fate of the dye could be followed in the live mice using a whole-animal imaging device [IVIS-200X (cy5.5 filter), Xenogen]. For all imaging, the mice were gently anesthetized with isoflurane and placed on a warm platform in the imaging device.

An aliquot of CVM in ethanol was mixed with phospholipid solvent for bath sonication preparation by the procedure described above. CVM labeled SapC-DOPS nanovesicles were separated from free CVM fluorophore using a Sephadex™ G25 column (PD-10, Amersham Pharmacia Biotech, Piscataway, NJ).

#### Bioluminescence of pancreatic tumor cells

To track tumor cells, luciferase expressing cfPac1-Luc3 human pancreatic tumor cells were used. After injection of luciferin, the same live imaging device (IVIS-200X, Xenogen) was used for visualization and localization of the bioluminescence.

### *In vitro* Studies

#### Effect of SapC-DOPS nanovesicles on tumor cells

Three widely used pancreatic adenocarcinoma cell lines (MiaPaCa-2, PANC-1, and BxPC-3) and untransformed cells (HPDE) were seeded (10^4^/100 µl/well) in 96-well flat-bottom tissue culture plates (Falcon, Becton Dickson Labware, Franklin Lakes, NJ) and cultured in their respective growth media for 24 hours, after which SapC-DOPS (0.14 mg) or PBS vehicle were added to the culture medium. To assess cell viability, a standard 3-(4,5-dimethylthiazol-2-yl)-2,5-diphenyltetrazolium bromide (MTT)-dye assay (Sigma-Aldrich, St. Louis, MO) was carried out as previously described [9], [36] three days after initiating treatment. Microscopic evaluation of tumor cells and HPDE cells was performed.

In order to evaluate for apoptosis, MiaPaCa-2 pancreatic cancer cells were treated with SapC-DOPS, PBS, or DNAase (positive control), and subsequently evaluated with terminal deoxynucleotidyl transferase-mediated dUTP nick end labeling (TUNEL) assay using In Situ Cell Death Detection Kit, POD (Roche Applied Science, Germany) as described in the manufacturer protocol.

In order to confirm that it was the assembled SapC-DOPS nanovesicles that caused cell death, MiaPaCa-2 pancreatic cancer cells and HPDE were treated with either SapC-DOPS, SapC alone, or DOPS alone. Cell viability was assessed with a MTT-dye assay.

To determine whether SapC-DOPS-induced apoptosis was mediated by caspase activation, proteins from nanovesicles-treated MiaPaCa-2 cells were analyzed by western blots. The cells were treated for 48 hours with SapC-DOPS, DOPS, PBS, or staurosporine (positive control) and then lysed for protein analysis by immunoblotting using NuPAGE Novex Bis-Tris Gels (4–12%), and the electrophoresis procedure per manufacturer (Invitrogen, Carlsbad, CA). Protein was transferred using a Semi-Dry blotting unit (FB-SDB-2020, Fisher Biotech, Pittsburgh, PA) to Hybond-ECL Nitrocellulose membrane (Amersham Biosciences, Piscataway, NJ). Anti-human caspase-9 and anti-actin antibodies (Cell Signaling Technology, Danvers, MA) were used for detection of active caspases and actin (control), respectively.

These experiments were performed in quadruplicate and data were analyzed by Analysis of Variance (ANOVA). T-test analysis or Two-way ANOVA Tukey test were used to determine statistical significance for experiments with two or greater than two groups, respectively. Analyses were done with SPSS 12.0.

#### Correlation between killing effect of SapC-DOPS and PS exposure on pancreatic cell surface

A flow cytometric analysis with FITC labeled Annexin V was performed on 8 cell lines (Hs766T, AsPC-1, cfPac1-Luc3, Capan-1, BxPC-3, MiaPaCa-2, HPAF-II, and PANC-1) to determine level of PS exposure on the cell surface. The cell lines were then separated into two groups: the “High-PS” group contained those that had a mean fluorescence (MF) over 50 (arbitrary units) and the “Low-PS” group contained those with a MF less than 50. The cells were treated with SapC-DOPS nanovesicles and the percentage of cell death was determined by MTT assay. MTT experiments were performed in quadruplicate and data were analyzed by ANOVA. The data presented are the arithmetic mean ± SEM.

### *In vivo* Studies

#### Ethics statement of animal use

All animal studies were approved by the Institutional Animal Care and Use Committee of the University of Cincinnati (IACUC Protocol Number: 11-05-05-02) and Cincinnati Children’s Hospital Medical Center (IACUC Protocol Number: 1E03031). Animals were anesthetized with isoflurane or pentobarbital for restraint before injections of tumor cells to minimize pain and distress. The depth of anesthesia was monitored by checking pain response (toe or tail pinch), muscle and jaw laxity, and the presence of regular respirations. Animals were euthanized when tumors reached 20% of body mass (i.e. size of the head), ulcerated or necrotized. Tumor-bearing animals were euthanized with carbon dioxide when normal physiological functions such as eating and drinking, urinating and defecating were impaired. The early removal criteria due to complications of surgery included discharge from site of surgery, loss of appetite, weight loss, and failure to groom. Early removal criteria as a result of tumor growth included hemiplegia, no response to stimuli such as noise or touch for a period greater than 12 hours after analgesic administration, weight loss greater than 20% with weekly weighing, dehydration, failure to groom, or lethargy for greater than 24 hours. These criteria were addressed by consultation with the attending veterinarian.

*In vivo* models were used to investigate the specificity, anti-tumor activity and sensitization enhanced cytotoxicity of SapC-DOPS nanovesicles in targeting the pancreatic tumor cells. For all *in vivo* studies, female nude/nude athymic mice (Taconic Farms, Germantown, NY, total 98) weighing approximately 20–25 grams were used. Tumor cells were either injected subcutaneously or orthotopically (Figure S1, Materials S1). All IV injections were performed in the tail vein. To confirm the presence of human pancreatic tumors, hematoxylin and eosin (H&E) staining of xenografted tumors was performed; examples are shown in Figure S2.

#### Dose assessment for inhibition of pancreatic tumor growth by SapC-DOPS

Mice (total 24) were inoculated subcutaneously in the right flank with a suspension of PANC-1 tumor cells (10^7^cells/mouse). Twenty-four days following inoculation, tumor size was measured using vernier calipers and the formula V = (π/6)LW^2^ (V = volume, L = length, W = width) [36]. The following day (day 1), groups of tumor-bearing mice (n = 6/group) were each injected intravenously with SapC-DOPS (1, 4, or 8 mg/kg at a 0.2 ml dose volume) or vehicle control (0.2 ml of PBS). Tumor size measurements were performed daily and injections were continued every other day until the largest tumors reached >2000 mm^3^ size (18 days). The mice were then sacrificed and an actual tumor weight was measured. Statistical analyses in this portion of the study were performed with GraphPad Prism® v4 software. Differences in final tumor weights were confirmed using ANOVA with Dunnett’s Multiple Comparison Post Test.

#### Evaluation of the ability of SapC-DOPS to target surface PS on pancreatic tumor cells

To determine whether SapC-DOPS nanovesicles specifically target pancreatic tumor cells *in vivo*, MiaPaCa-2 cells (5×10^6^ cells) were injected subcutaneously into the flanks of the mice. After 5 weeks, when the tumors were palpable, one of the following was IV injected into each mouse: fluorescently labeled SapC-DOPS nanovesicles (6 mg/kg), non-complexed SapC and fluorescently labeled DOPS, fluorescently labeled DOPS alone, or PBS control (5 mice/group, total 20). Assessment of the *in vivo* distribution of fluorescence was performed with the live-animal imaging device. Images were taken at multiple time intervals from 5 minutes to 100 hours post-injection.

To determine the blocking effect of PS binding, luciferase expressing cfPac1-Luc3 pancreatic tumor cells were used. The cells were pretreated with the PS binding proteins lactadherin-C2 [41] and β2-glycoprotein [42] (0.1 mg/ml), or left in medium without treatment as a control for one hour and then injected subcutaneously on the top of the head of nude mice (total 8). Bioluminescence imaging was performed to visualize the tumor cells. At one hour post-implantation, fluorescently labeled SapC-DOPS nanovesicles were IV injected, and fluorescence signal was documented using the live imaging system.

To investigate SapC-DOPS clearance from normal mouse tissue, fluorescently labeled SapC-DOPS was IV injected into 5 mice. The mice were then sacrificed and the liver, spleen, lung, heart, and kidney were removed and imaged at 1, 12 and 24 hours after injection to evaluate for the presence of fluorescently labeled SapC-DOPS.

#### Effect of SapC-DOPS nanovesicles on pancreatic tumor growth

MiaPaCa-2 pancreatic tumor cells (5×10^6^ cells), which we documented in the *in vitro* studies were susceptible to SapC-DOPS treatment (>80% cell death), were injected subcutaneously in the upper back of mice. Tumor size was measured with calipers every 3 days and volume was calculated. When the mean tumor volume grew to ∼500 mm^3^, the mice were IV injected with either SapC-DOPS (6 mg/kg) or PBS alone (5 mice/group, total 10). Injections were repeated on day 3, 6, 12, 15, 18, 21, and 24, and tumor measurements were continued until day 30. T-test analysis or Two-way ANOVA Tukey test were used to determine statistical significance for experiments with two or greater than two groups, respectively. Analyses were done with SPSS 12.0.

#### Tracking of tumor bioluminescence and SapC-DOPS fluorescence in mice with orthotopic pancreatic tumors

To demonstrate how SapC-DOPS selectively targets orthotopic pancreatic tumors, human cfPac1-Luc3 cells were injected into the pancreas of the nude mouse. After an orthotopic pancreatic tumor was detected using live imaging at day 6, fluorescently labeled SapC-DOPS nanovesicles, fluorescently labeled SapC, fluorescently labeled DOPS, or PBS (control) were IV injected (6 mice/group, total 24).

#### Survival of SapC-DOPS-treated mice with orthotopically implanted pancreatic tumors

Groups of mice (n = 6 each, total 12) with orthotopically injected pancreatic tumor cells (luciferase-expressing cfPac1-Luc3 line) confirmed by bioluminescence on live imaging were IV injected with multiple doses (day 6, 9, 12, 15, 19, 23, 27, 30, 34, 41) of SapC-DOPS (6 mg/kg in 30 µL PBS) or vehicle control (PBS 30 µL). The fluorescently labeled SapC-DOPS nanovesicles were then detected using the live imaging system. The surviving animals were monitored until all mice in the control group expired. Survival curves were created using the Kaplan and Meier method with GraphPad Prism software. The bioluminescent tumors were monitored with the live imaging device for 23 weeks.

#### Long-term tracking of tumor bioluminescence and SapC-DOPS fluorescence in mice with orthotopic pancreatic tumors

Luciferase-expressing cfPac1-Luc3 pancreatic tumor cells were orthotopically injected in the mouse pancreas. After 110 days, the distribution of bioluminescent tumors was monitored using the live-animal imaging device. After the bioluminescence dissipated, fluorescently labeled SapC-DOPS nanovesicles were injected (6 mg/kg) and the mice were imaged again.

#### Targeting of concealed orthotopically-implanted and metastatic tumors by fluorescently-labeled SapC-DOPS nanovesicles

Orthotopic pancreatic tumors were generated by implanting a luciferase-expressing pancreatic tumor line (cfPac1-Luc-3) in mice. After 6 weeks, the mice were imaged to identify all tumor sites. Once the bioluminescence had dissipated, fluorescently labeled SapC-DOPS was IV injected.

## Results

### *In vitro* Studies

#### Effect of SapC-DOPS nanovesicles on tumor cells

**Figure 1:**
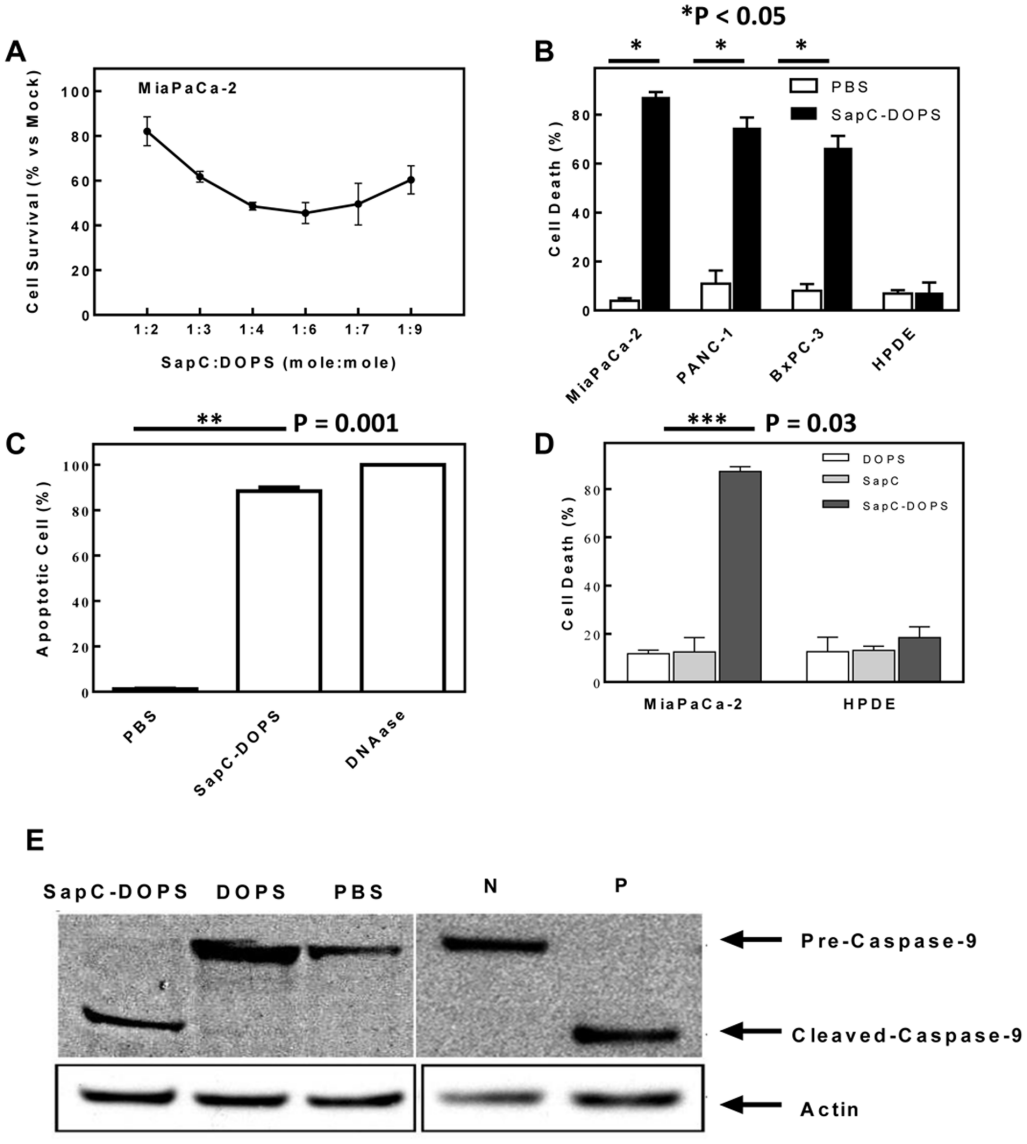
Killing effect of SapC-DOPS nanovesicles on pancreatic tumor cells *in vitro*. (A) Role of SapC and DOPS molar ratio for cytotoxicity on human pancreatic (MiaPaCa-2) cancer cells. (B) Cell death (%) in human pancreatic cancer cells (MiaPaCa-2, PANC-1, and BxPC-3) and normal controls (HPDE) after exposure to SapC-DOPS. (Data in 1B–1D are reported as arithmetic mean ± SEM.) (C) Apoptosis (%) in MiaPaCa-2 pancreatic cancer cells following treatment with either SapC-DOPS, PBS (negative control), or DNAase (positive control). (D) Cell death (%) in human pancreatic cancer cells and HPDE after exposure to SapC-DOPS, SapC alone, or DOPS alone. (E) Western blot analysis demonstrates that treatment of MiaPaCa-2 pancreatic cancer cells with both the SapC-DOPS and staurosporine positive control (P) resulted in cleavage of pre-caspase-9 to active caspase-9, while treatment with DOPS, PBS, and negative control (N) did not cause enzyme activation.

**Figure 2:**
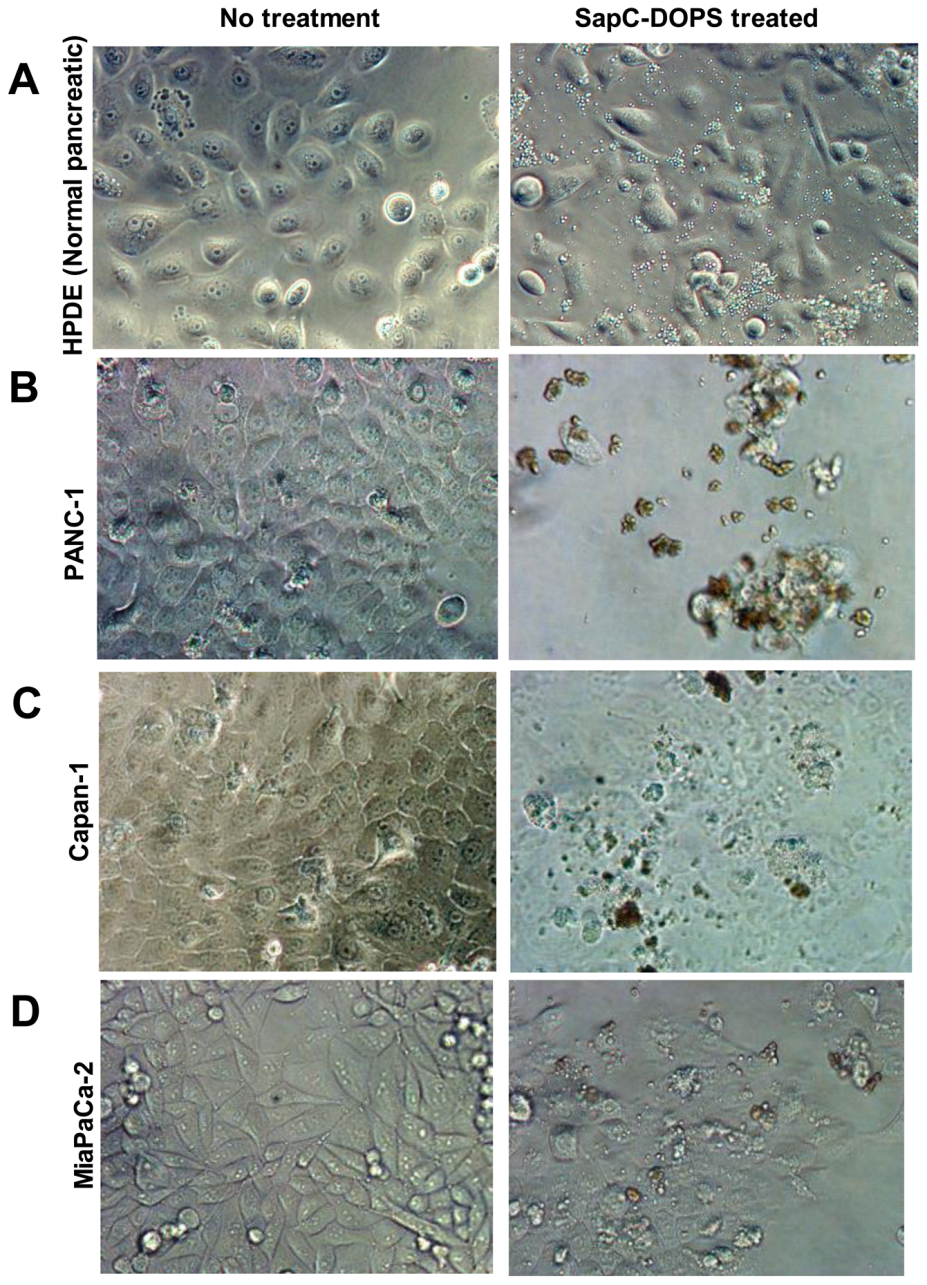
Microscopic inspection of cells with and without SapC-DOPS treatment. The tumor cells (PANC-1, Capan-1 and MiaPaCa-2 in (B), (C), and (D), respectively) had morphological features consistent with apoptotic death after treatment with SapC-DOPS, while the untransformed HPDE cells (A) appeared normal.

In previous studies, we found that the optimal molar ratio of SapC and DOPS was from 1∶3 to 1∶10 for maximal cytotoxic effect against human neuroblastoma and skin cancer cells [36], [37]. We determined that this molar range of SapC:DOPS ratio also had significant killing effect on human pancreatic (MiaPaCa-2) cancer cells (Figure 1A). When the three pancreatic adenocarcinoma cell lines and HPDE cells were treated with SapC-DOPS, most of the tumor cells died, while the HPDE cells remained viable (Figure 1B). Microscopic inspection of SapC-DOPS-treated cells indicated that the tumor cells had morphological features consistent with apoptotic death, while the untransformed HPDE cells appeared normal (Figure 2). TUNEL analysis revealed that SapC-DOPS did indeed induce apoptosis (Figure 1C). Unlike SapC-DOPS and the positive control DNAase, the negative PBS control had no apoptotic effect. Both components of SapC-DOPS were essential for optimal tumor cell killing. If just the protein (SapC) or the phospholipid (DOPS) components were individually added to cells, there was no induction of apoptosis and the percentage of apoptotic cells was comparable to the PBS control (Figures 1B and 1D). Western blot analysis revealed that cleavage of the proenzyme pre-caspase-9 to the active form occurred only in the SapC-DOPS-treated cells and the staurosporine-treated cells (Figure 1E).

#### Correlation between killing effect of SapC-DOPS and PS exposure on pancreatic cell surface

**Figure 3:**
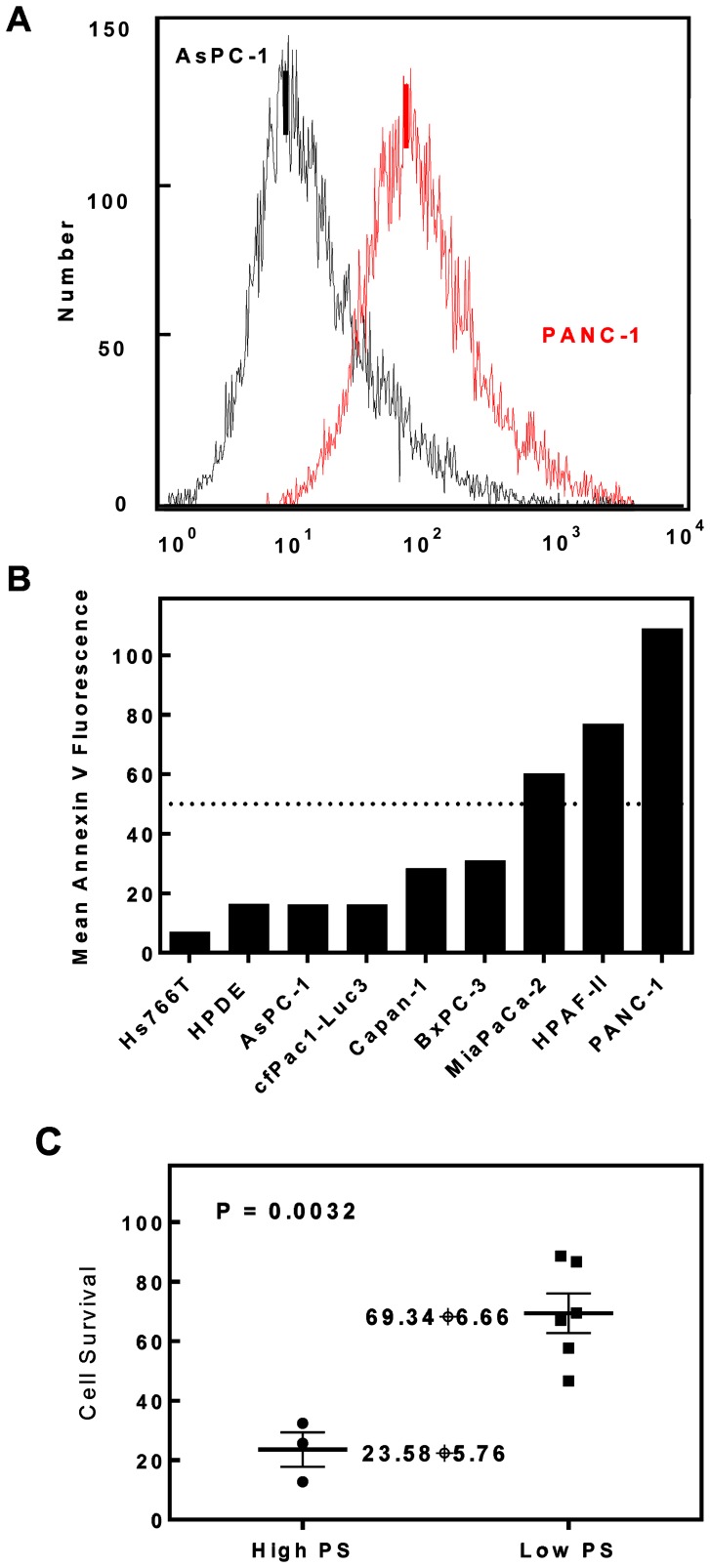
Correlation between SapC-DOPS killing effect and pancreatic cell surface PS exposure. (A) Fluorescence histogram of PS levels on PANC-1 and AsPC-1 cell surfaces measured by Annexin V-FITC. (B) The differential PS expression on human pancreatic cell surface of 9 cell lines. (C) Combined percentage of cell survival in the two groups of cell lines treated with SapC-DOPS as determined by MTT assay (p = 0.0032).

The fluorescence histogram in Figure 3A demonstrates that the external PS level on PANC-1 cells was higher than for AsPC-1 cells. The relative PS expression on the cell surface of human pancreatic tumor cell lines is demonstrated in Figure 3B. Among these cell lines, MiaPaCa-2, HPAF-II, and PANC-1 had MF over 50 and therefore comprised the “High-PS” group. As shown in Figure 3C, the cells in the High-PS group demonstrated significantly greater cytotoxicity in response to SapC-DOPS than the cells in the Low-PS group (p<0.05).

### *In vivo* Studies: Subcutaneous Pancreatic Tumor Model

#### Dose assessment for inhibition of pancreatic tumor growth by SapC-DOPS

**Figure 4:**
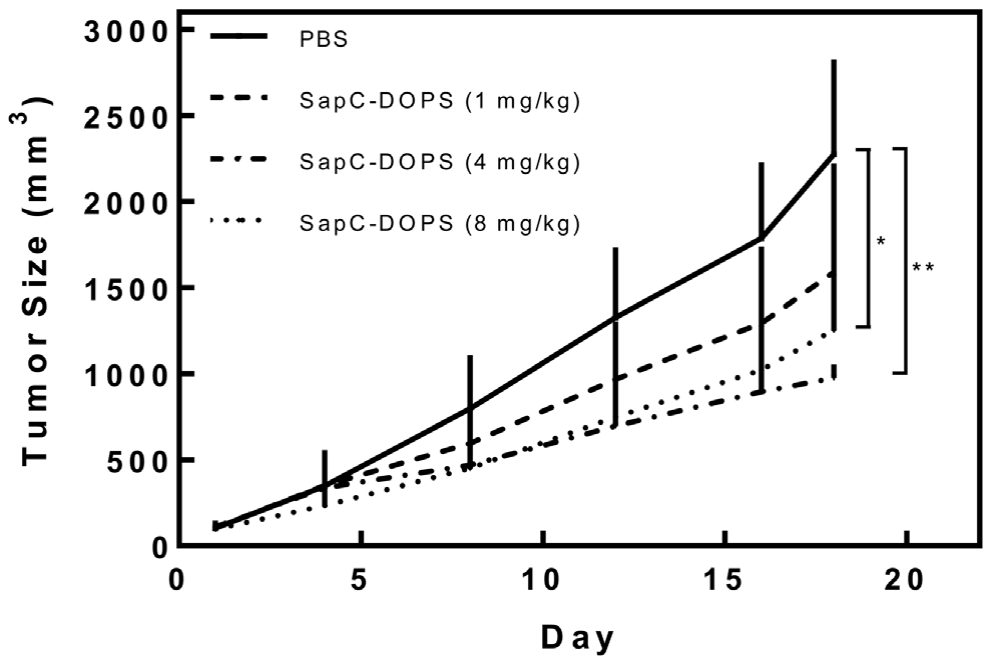
Dose-dependent inhibition of pancreatic tumor growth by SapC-DOPS in xenograft model. PANC-1 xenograft tumors in nude mice were treated every other day with 1, 4, or 8 mg/kg of SapC-DOPS, or with PBS control. Tumor sizes at high doses (4 mg/kg and 8 mg/kg) were significantly smaller than control and 1 mg/kg groups (p = 0.003* and 0.00015**, respectively).

Tumor sizes after administration of SapC-DOPS at doses of 4 mg/kg and 8 mg/kg every other day were significantly smaller than that of the control group and 1 mg/kg treatment group (p = 0.003* and 0.00015**) after 18 days of treatment (Figure 4). Based on these data, we chose a treatment dose of 6 mg/kg for efficacy studies.

#### Evaluation of the ability of SapC-DOPS to target surface PS on pancreatic tumor cells

**Figure 5:**
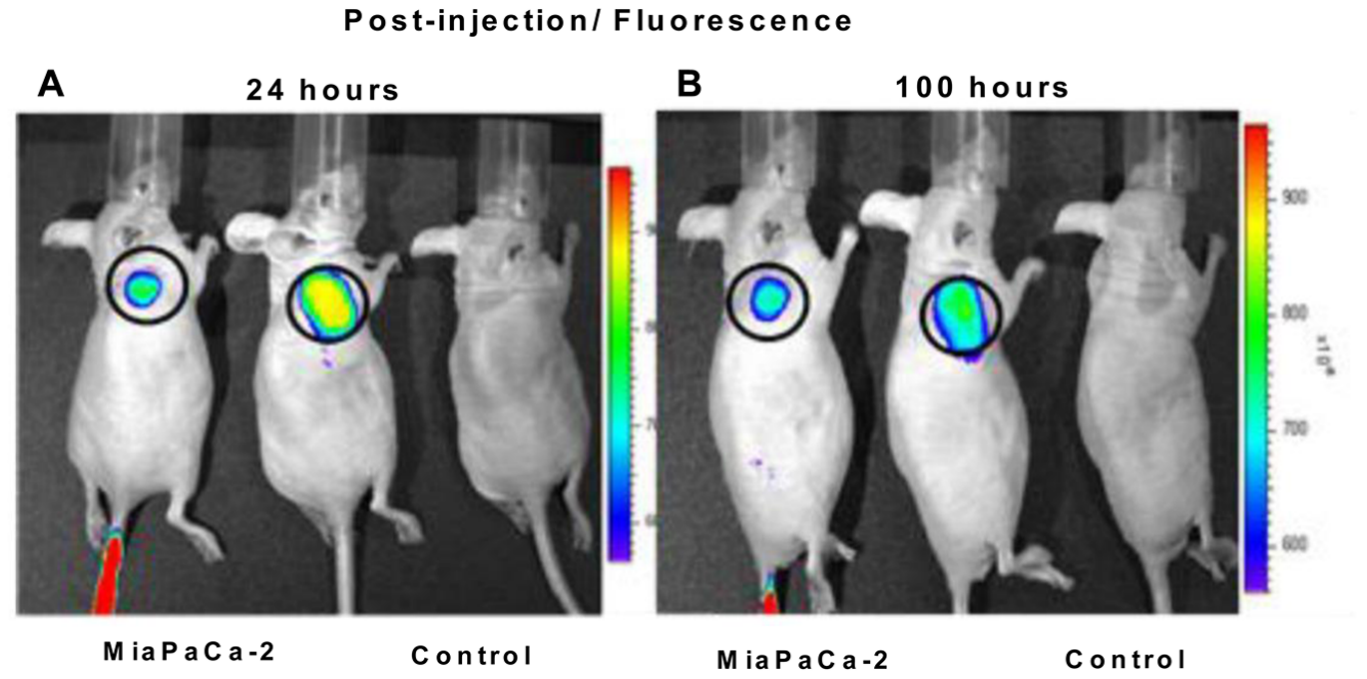
Targeting of fluorescently-labeled SapC-DOPS nanovesicles to pancreatic tumor cells *in vivo*. The nanovesicles were rapidly targeted to the palpable tumor within 5 minutes (data not shown). The fluorescence persisted for over 1 (A) and 4 (B) days. SapC = 3.2 mg/kg, DOPS = 1.8 mg/kg, CVM = 6 µM.

**Figure 6:**
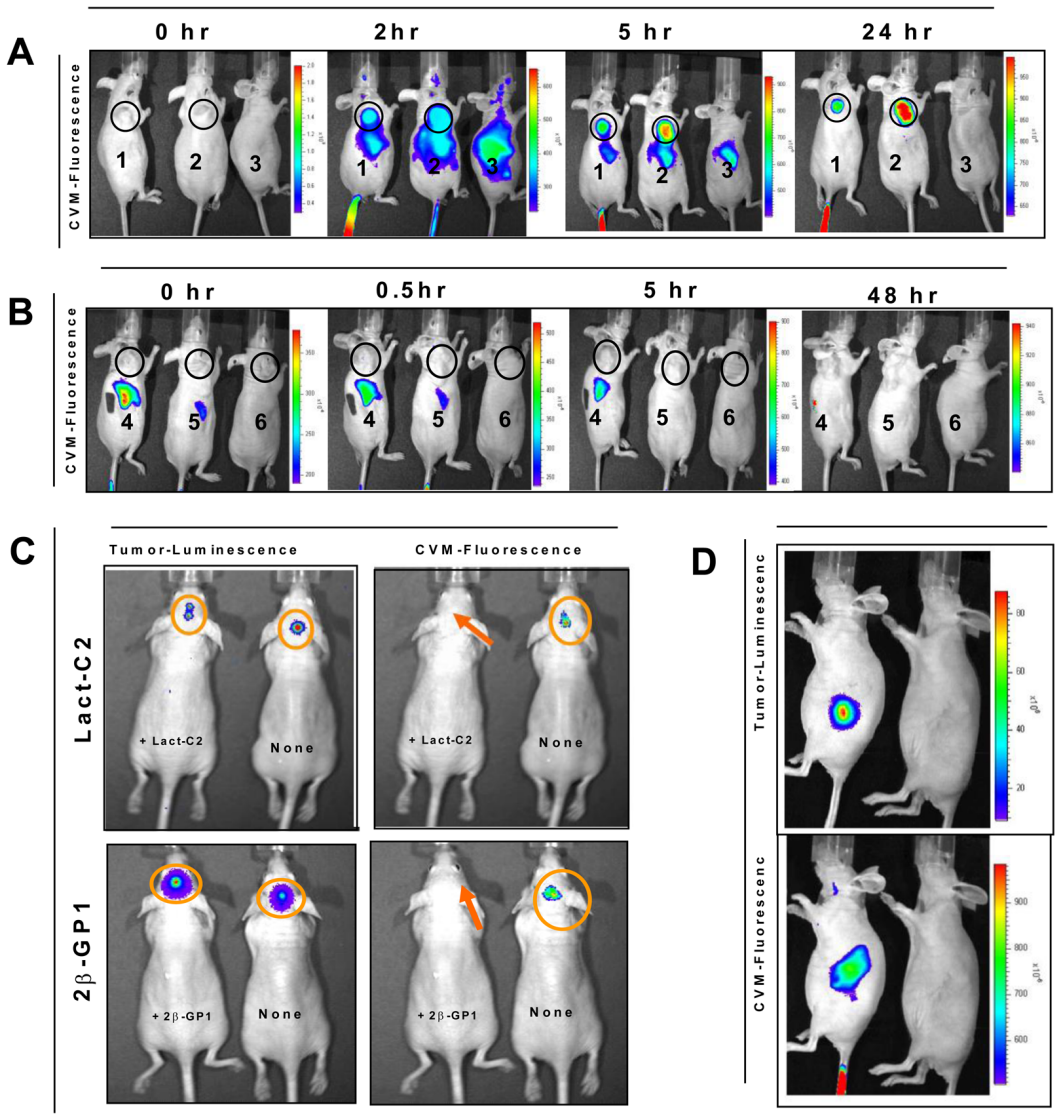
Targeting of subcutaneous tumor xenografts by fluorescently labeled SapC-DOPS nanovesicles. Heterotopic MiaPaCa-2 tumors (circled) were generated by subcutaneous injection in the upper flank of nude mice. (A) Mice 1 & 2 were tumor-bearing mice, injected with fluorescently labeled SapC-DOPS nanovesicles; Mouse 3 was non-tumor-bearing, PBS injected. Mice 1 and 2 both demonstrate localization of the fluorescent label to the tumor site. Fluorescence also localizes to the liver by 2 hours, but is gone by 24 hours. (B) Tumor-bearing mice 4, 5, and 6 were injected with non-complexed SapC and fluorescently labeled DOPS, fluorescently labeled DOPS only, and PBS, respectively. There is no fluorescence localized to tumor in any of these mice. Like the SapC-DOPS (in mice 1 and 2), non-complexed SapC and fluorescently labeled DOPS and fluorescently labeled DOPS alone (in mice 4 and 5, respectively) did localize to the liver, but quickly dissipated (C) Subcutaneous tumors created using cfPac1-Luc3 pancreatic tumor cells that were and were not pretreated with PS-specific binding proteins (Lactadherin-C2 [left upper panel] and Beta-GP-1 [left lower panel]) display bioluminescence on live imaging. After administration of CVM fluorescently labeled SapC-DOPS nanovesicles, the tumors that were not pretreated demonstrated fluorescence, while the tumors that had been pretreated did not demonstrate any fluorescence (right upper and lower panels). (D) Presence of bioluminescence (upper panel) confirms presence of orthotopic pancreatic tumor, and co-localized fluorescence (bottom panel) confirms targeting by fluorescently labeled SapC-DOPS nanovesicles.

Live imaging demonstrated that fluorescently labeled SapC-DOPS nanovesicles were rapidly targeted to the palpable tumor within 5 minutes (data not shown) and the fluorescence persisted for over 4 days (Figure 5). The fluorescence from the assembled SapC-DOPS nanovesicles targeted to the tumor sites, whereas no tumor fluorescence was noted after co-injection of non-complexed SapC and fluorescently labeled DOPS, or after injection of fluorescently labeled DOPS alone or PBS control (Figures 6A and 6B). While fluorescently labeled SapC-DOPS was noted to accumulate in the liver early (2 hours after injection), there was no trace of liver fluorescence on live imaging after 24 hours (Figure 6A). Similarly, the non-complexed SapC and fluorescently labeled DOPS, and the fluorescently labeled DOPS alone, were identified in the liver at 0.5 hours on live imaging, but quickly dissipated (Figure 6B).

When human tumor cells (cfPac1-Luc3) pretreated with Lact-C2 or Beta-GP-1 were injected subcutaneously, followed one hour later by administration of intravenous fluorescently labeled SapC-DOPS nanovesicles, no fluorescence signal was identified at the tumor cell site (Figure 6C). Conversely, fluorescence was detected at the tumor cell location in animals with tumor cells that were not pretreated with Lact-C2 or Beta-GP-1 (Figure 6C). This indicates that both Lact-C2 and Beta-GP-1 completely block cell surface PS lipids from SapC-DOPS binding.

**Figure 7:**
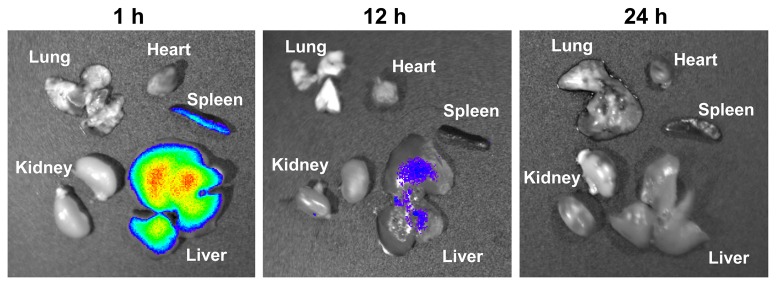
Localization of fluorescently labeled SapC-DOPS in normal mouse tissue. The organ distribution of fluorescently labeled SapC-DOPS 1, 12 and 24 hours after injection is shown in (A), (B), and (C) respectively. The liver and spleen were the only organs that had detectable fluorescent signal 1 and 12 hours post-injection for all animals. This signal was no longer present by 24 hours post-injection. The lung, heart, and kidney showed no detectable fluorescent signal. SapC = 3.2 mg/kg, DOPS = 1.8 mg/kg, CVM = 6 µM.

On specific organ examination, the liver and spleen were the only two organs that had detectable fluorescent signal 1 and 12 hour post-injection for all animals. This signal was no longer present by 24 hours post-injection (Figure 7).

#### Effect of SapC-DOPS nanovesicles on subcutaneous pancreatic tumor growth

**Figure 8:**
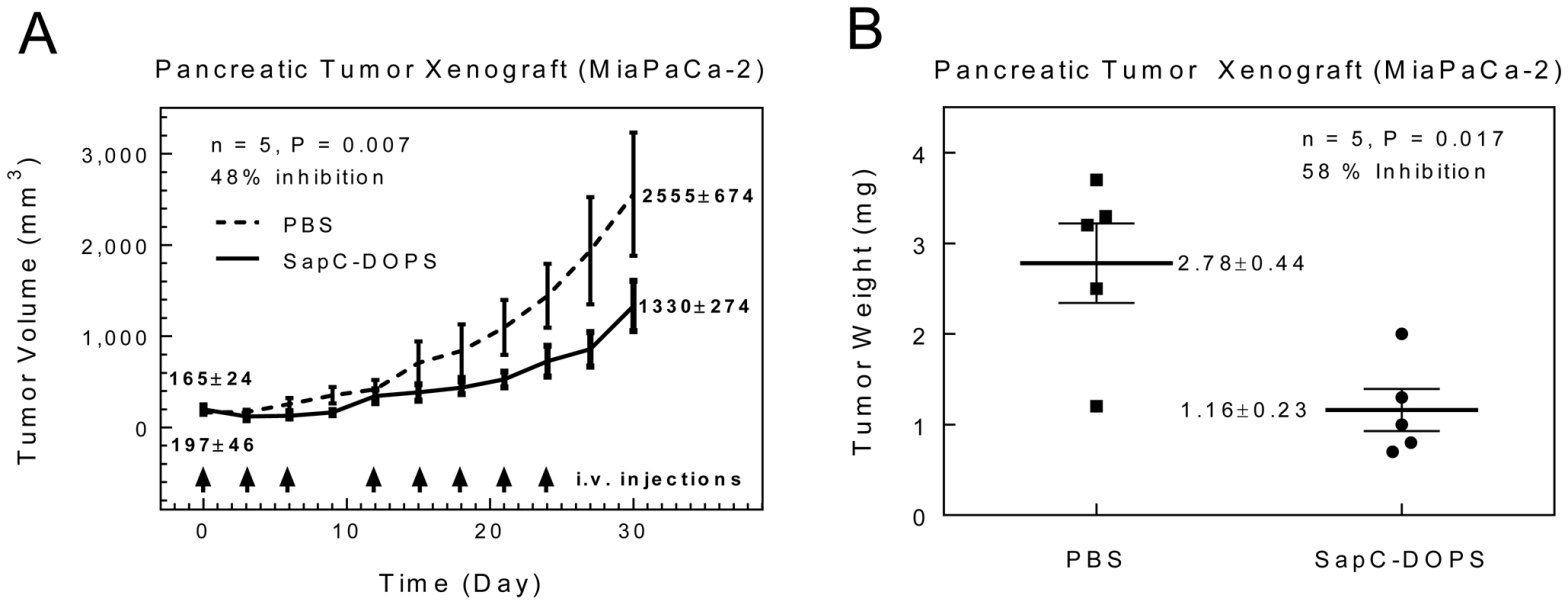
Effect of SapC-DOPS nanovesicles on pancreatic tumor growth. Subcutaneous tumors (MiaPaCa-2 cell line) treated with SapC-DOPS nanovesicles had significantly reduced tumor end-volume (A) and end-weight (B) compared to controls.

When the mice with subcutaneous tumors of approximately 500 mm^3^ size were injected with SapC-DOPS or PBS (control), the treated group had a mean reduction in tumor end-volume and end-weight of 48% and 58%, respectively (Figures 8A and 8B). The difference in end-volume and end-weight between the treatment and control groups was significant (p = 0.007 and 0.017, respectively).

### *In vivo* Studies: Orthotopic Pancreatic Tumor Model

#### Tracking of tumor bioluminescence and SapC-DOPS fluorescence in mice with orthotopic pancreatic tumors

When fluorescently labeled nanovesicles were injected into mice with orthotopic pancreatic tumors, the fluorescence co-localized with the bioluminescent tumors (Figure 6D). Neither DOPS nor SapC alone with CVM had tumor targeting ability (data not shown).

#### Survival of SapC-DOPS-treated mice with orthotopically implanted pancreatic tumors

**Figure 9:**
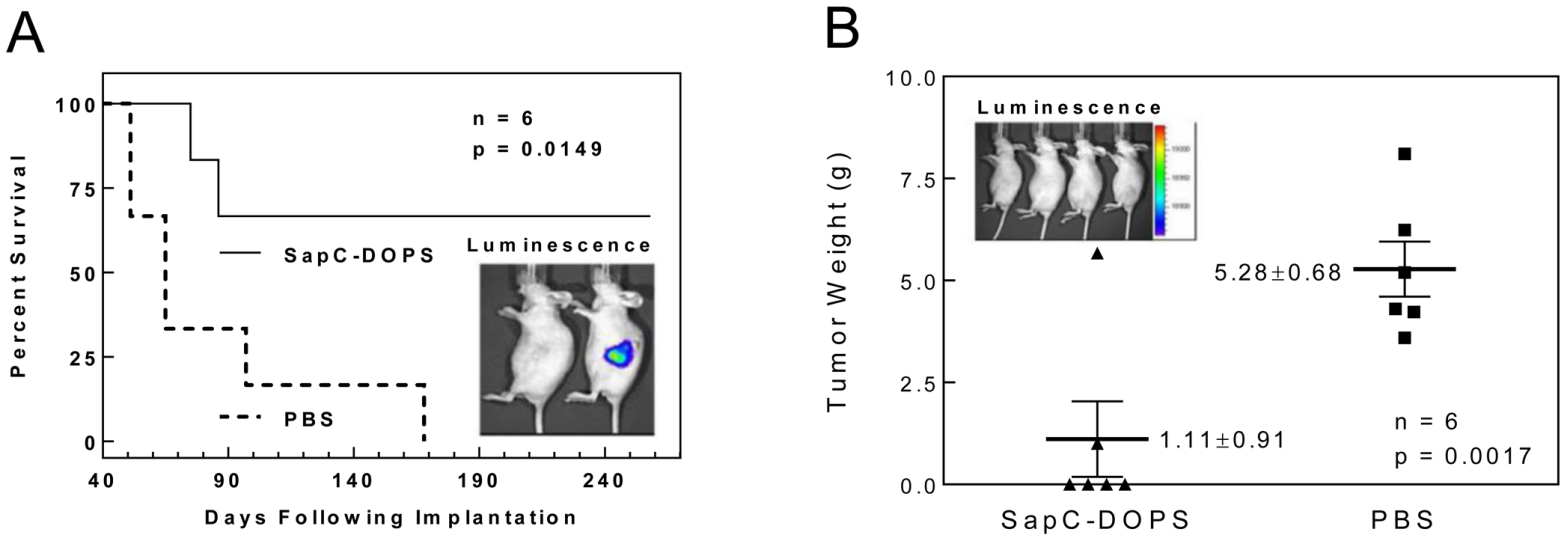
Survival of SapC-DOPS-treated mice with orthotopically implanted pancreatic tumors. (A) Survival of SapC-DOPS-treated mice was significantly greater than controls. Insert panel: bioluminescence confirms hidden implanted pancreatic tumor on live imaging (mouse on right). (B) Tumor weight at time of death is shown. No tumor was noted in the four surviving mice at post-treatment day 110, as documented by the absence of bioluminescence in those mice (insert panel).

Mice with confirmed pancreatic tumors that were treated with SapC-DOPS lived significantly longer than controls (p = 0.0149) (Figures 9A and 9B). After 23 weeks, all control mice had died; 4/6 SapC-DOPS-treated mice were still alive.

#### Long-term tracking of tumor bioluminescence and SapC-DOPS fluorescence in mice with orthotopic pancreatic tumors

When mice with orthotopically implanted cfPac1-Luc3 pancreatic tumors that were treated with SapC-DOPS were assessed for bioluminescence 110 days following implantation, no bioluminescence was detected, indicating complete resolution of tumors (Figure 9B, insert).

#### Targeting of concealed orthotopically-implanted and metastatic tumors by fluorophore-labeled SapC-DOPS nanovesicles

**Figure 10:**
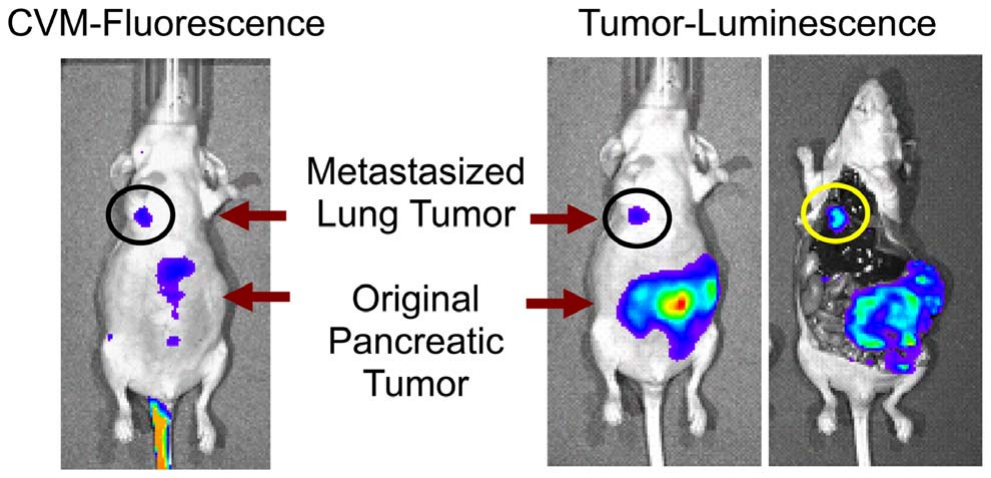
Targeting of primary pancreatic and metastatic lung tumors by SapC-DOPS. Co-localization of CVM-florescence and tumor-bioluminescence in both metastatic lung tumor and primary pancreatic tumor. Tissue dissection confirmed that fluorescence was tumor-associated. SapC = 3.2 mg/kg, DOPS = 1.8 mg/kg, CVM = 6 µM.

After 6 weeks of growth of orthotopically-implanted pancreatic tumor, bioluminescence signal was detected in the lung of one mouse, which suggested that primary pancreatic cells had metastasized to the lung (Figure 10). Within hours of injection of the fluorescently labeled SapC-DOPS, co-localization of the fluorescence with the bioluminescence was evident in the metastatic lung tumor. Upon tissue dissection, lung tumor was positively identified, and fluorescence was confirmed to be tumor-associated.

## Discussion

**Figure 11:**
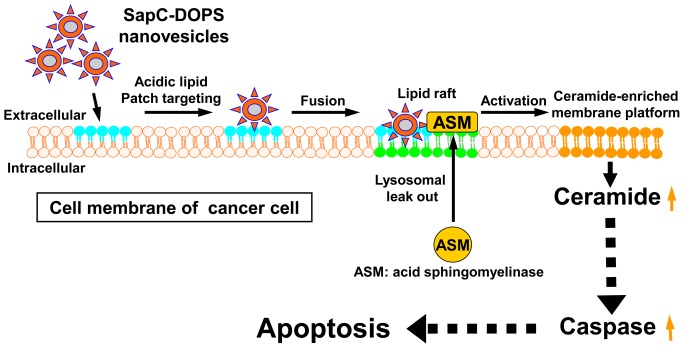
Hypothetical diagram of cancer-selective targeting and apoptosis induction by SapC-DOPS nanovesicles.

Because pancreatic tumors are typically discovered at very late stages and therapeutic intervention is usually ineffective, there is an urgent demand for more efficacious treatments. To address this need, we are reporting a promising novel biotherapeutic agent composed of two natural cellular components that spontaneously assemble into stable nanovesicles (SapC-DOPS). Based on our current results, SapC-DOPS nanovesicles selectively attack pancreatic cancer by binding to tumor cells and inducing cell death. Though the molecular mechanism of tumor entry is not entirely clear, SapC-DOPS nanovesicles are thought to enter the tumor mass and bind to aberrantly exposed PS rich membrane domains that have been widely reported in cancer cells and cells of the tumor neovasculature. The SapC-DOPS nanovesicles may trigger acid sphingomyelinase (ASM)-derived ceramide signaling cascades to induce apoptosis of cancer cells, as we show in our hypothetical diagram in Figure 11. Susceptibility of cells to apoptosis is influenced by expression of a variety of genes, and may especially be altered during transformation.

Our previous report supported the notion that SapC–DOPS kills cancer cells, at least in part, through the accumulation of ceramides, leading to caspase activation and apoptotic cell death [36]. While our current *in vitro* evidence strongly indicates that SapC-DOPS induces caspase-mediated apoptotic cell death of human pancreatic cancer cells in culture, the molecular mechanism of tumor cell entry and caspase activation remain unknown. We showed that SapC-DOPS related cytotoxicity was much greater in pancreatic tumor cell lines that have greater PS expression on the cell surface. However, different cells lines with the same amount of external PS may vary in their response, as PS levels are likely not the sole determinant of effectiveness for SapC-DOPS treatment. The specific destruction of pancreatic cancer cells, but not normal pancreatic cells, in the *in vitro* studies prompted us to explore whether the nanovesicles might have similar anti-tumor activity *in vivo*.

Our studies using fluorescently labeled SapC-DOPS confirmed that the nanovesicles target pancreatic cell tumors *in vivo*, both in subcutaneous and orthotopic tumor models. However, the targeting of the nanovesicles (fluorescence signal) to the tumors was blocked after pretreatment with PS-specific binding proteins, indicating that SapC-DOPS binds with cell surface PS lipids. The SapC-DOPS nanovesicles also targeted pancreatic cell tumor metastases, as documented by fluorescence signal. Although fluorescently labeled SapC-DOPS was detected in the mouse liver and spleen, it disappeared by 24 hours, whereas it was able to be detected in subcutaneous pancreatic cell tumors for over 4 days.

We demonstrated significant inhibition of pancreatic tumor xenograft growth by SapC-DOPS nanovesicles as compared to control, and significantly increased survival of mice with orthotopically-implanted pancreatic tumors when treated with SapC-DOPS as compared to control. Both of these reflect the significant tumor-killing effect of SapC-DOPS.

Through these studies, we demonstrate that SapC-DOPS nanovesicles are a promising technology that selectively target pancreatic cancer cells, but not normal pancreatic cells. The reason for this targeting is likely threefold. First, SapC-DOPS nanovesicles directly interact and fuse with the exposed PS rich microdomains on cancer cell surface. Secondly, the acidic microenvironment that favors the activity of SapC is available only around the cancer cells. Thirdly, normal cells do not have as much sphingomyelinase, which results from pancreatic cancer cell membrane “leakiness.” The absence of toxicity and adverse side effects with use of SapC-DOPS nanovesicles [36], as well as their rapid clearance from normal tissues and accumulation only in pancreatic tumor tissues, provides significant evidence of their safety. Continued evidence of efficient tumor targeting and killing should prompt testing against other deadly cancers.

In our future studies, we plan to investigate the molecular mechanism of SapC-DOPS targeting using several approaches, including RNAseq to investigate relevant genes, western blots to determine protein expression level, and cell lysates using mass spectroscopy to confirm dose-dependent killing pathways. The safety of SapC-DOPS will need be tested on other animal models as we prepare to proceed with further clinical testing of this new treatment. Although no significant side effects were evidenced in our previous acute and chronic toxicology studies of SapC-DOPS nanovesicles in mice [36], we have not excluded the possibility of long-term adverse effects, such as a prothrombogenic reaction.

In summary, we report a therapeutic nanovesicle approach that uses a unique combination of the recombinant SapC and the phospholipid DOPS. Our data suggest that this combination selectively binds and fuses with aberrantly externalized acidic phospholipid PS patches on cancer cells and induces cell death. Further experiments will unveil more details about this proposed mechanism.

## Supporting Information

Figure S1 **Orthotopic pancreatic tumor cell injection techniques.** (A) A small left abdominal flank incision was made and the spleen exteriorized. (B) A successful subcapsular intrapancreatic injection of tumor cells was identified by the appearance of a fluid bleb without intraperitoneal leakage.
(TIF)

Figure S2 **H & E staining of xenografted human pancreatic tumors.** (A), (B), (C) and (D) show the xenografts of MiapaCa-2, PANC-1, BxPC-3 and cfPac1-Luc3, respectively, using 400× magnification.
(TIF)

Materials S1 (DOCX)
