# Correction: Targeting and Cytotoxicity of SapC-DOPS Nanovesicles in Pancreatic Cancer

**DOI:** 10.1371/journal.pone.0118232

**Published:** 2015-03-05

**Authors:** The PLOS ONE Staff

Fig. 1 is incorrect. The Western blot in Fig. 1E displays the incorrect cell line. The authors have provided a corrected version here.

**Fig 1:**
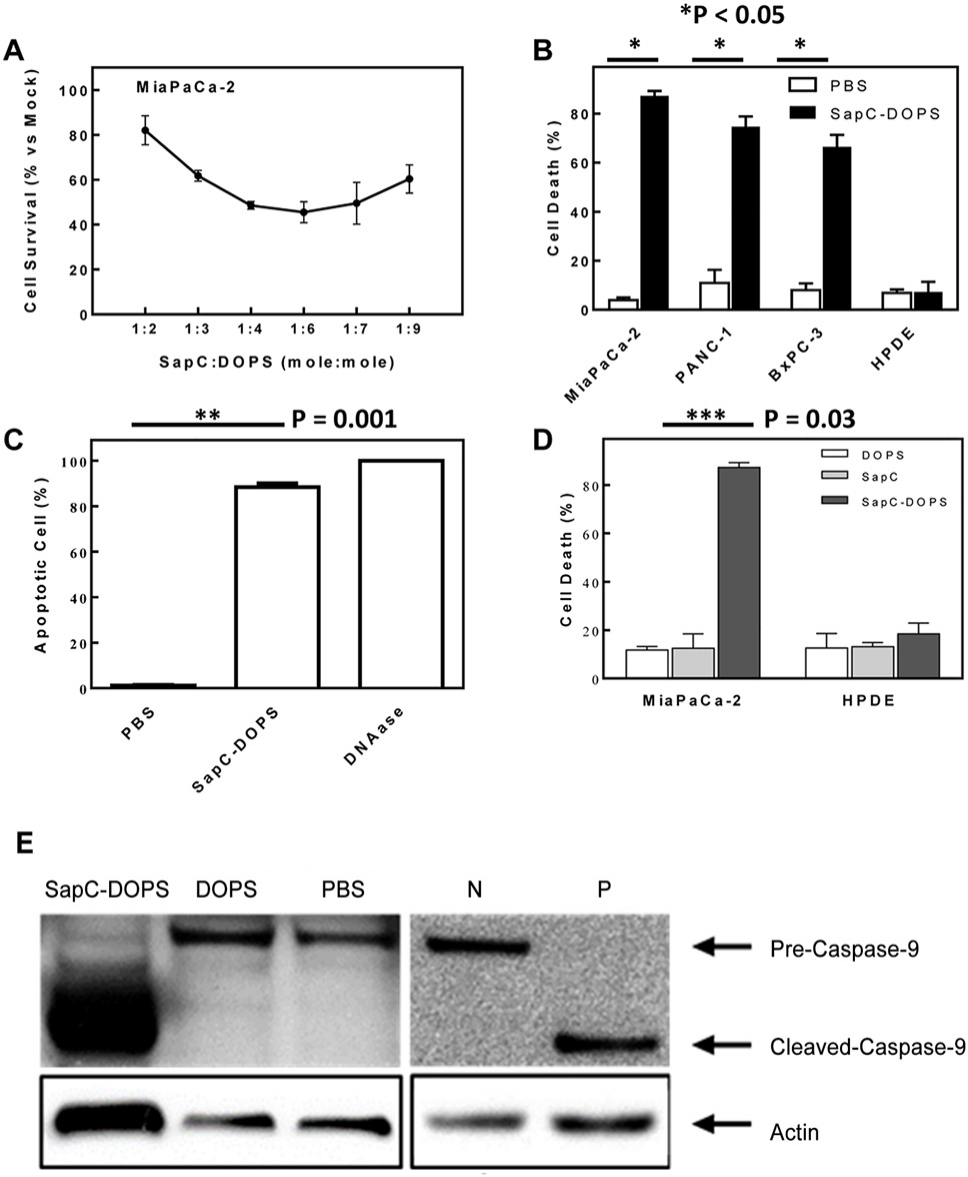
Killing effect of SapC-DOPS nanovesicles on pancreatic tumor cells *in vitro*. (A) Role of SapC and DOPS molar ratio for cytotoxicity on human pancreatic (MiaPaCa-2) cancer cells. (B) Cell death (%) in human pancreatic cancer cells (MiaPaCa-2, PANC-1, and BxPC-3) and normal controls (HPDE) after exposure to SapC-DOPS. (Data in 1B–1D are reported as arithmetic mean ± SEM.) (C) Apoptosis (%) in MiaPaCa-2 pancreatic cancer cells following treatment with either SapC-DOPS, PBS (negative control), or DNAase (positive control). (D) Cell death (%) in human pancreatic cancer cells and HPDE after exposure to SapC-DOPS, SapC alone, or DOPS alone. (E) Western blot analysis demonstrates that treatment of MiaPaCa-2 pancreatic cancer cells with both the SapC-DOPS and staurosporine positive control (P) resulted in cleavage of pre-caspase-9 to active caspase-9, while treatment with DOPS, PBS, and negative control (N) did not cause enzyme activation.
