# Supplementary figures and images for: Targeting and Cytotoxicity of SapC-DOPS Nanovesicles in Pancreatic Cancer
Source: PLoS One. 2013 Oct 4;8(10):e75507. doi: 10.1371/journal.pone.0075507 (PMC3790873; doi:10.1371/journal.pone.0075507)

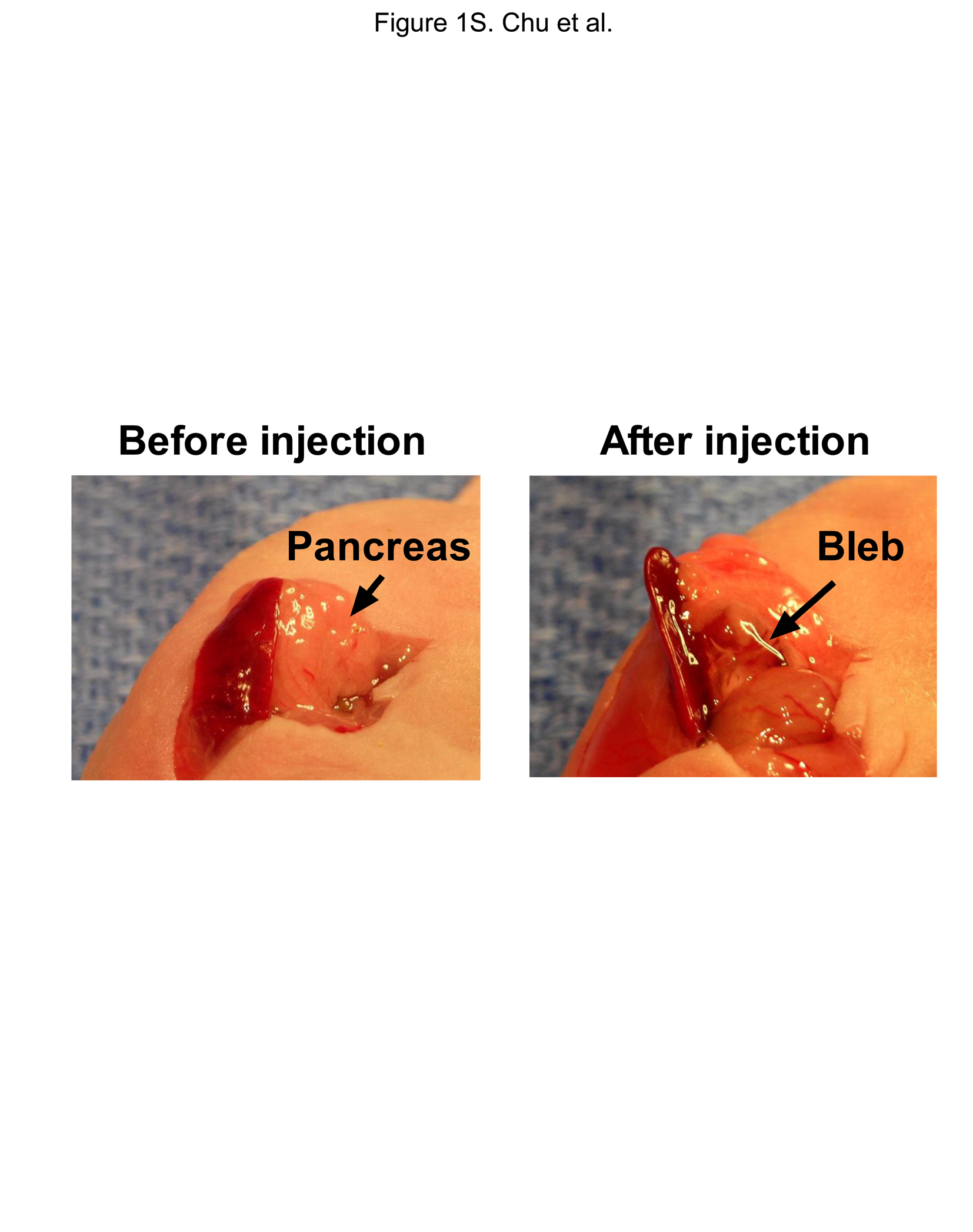

Supplement: Figure S1 — Orthotopic pancreatic tumor cell injection techniques. (A) A small left abdominal flank incision was made and the spleen exteriorized. (B) A successful subcapsular intrapancreatic injection of tumor cells was identified by the appearance of a fluid bleb without intraperitoneal leakage. (TIF) [file pone.0075507.s001.tif]

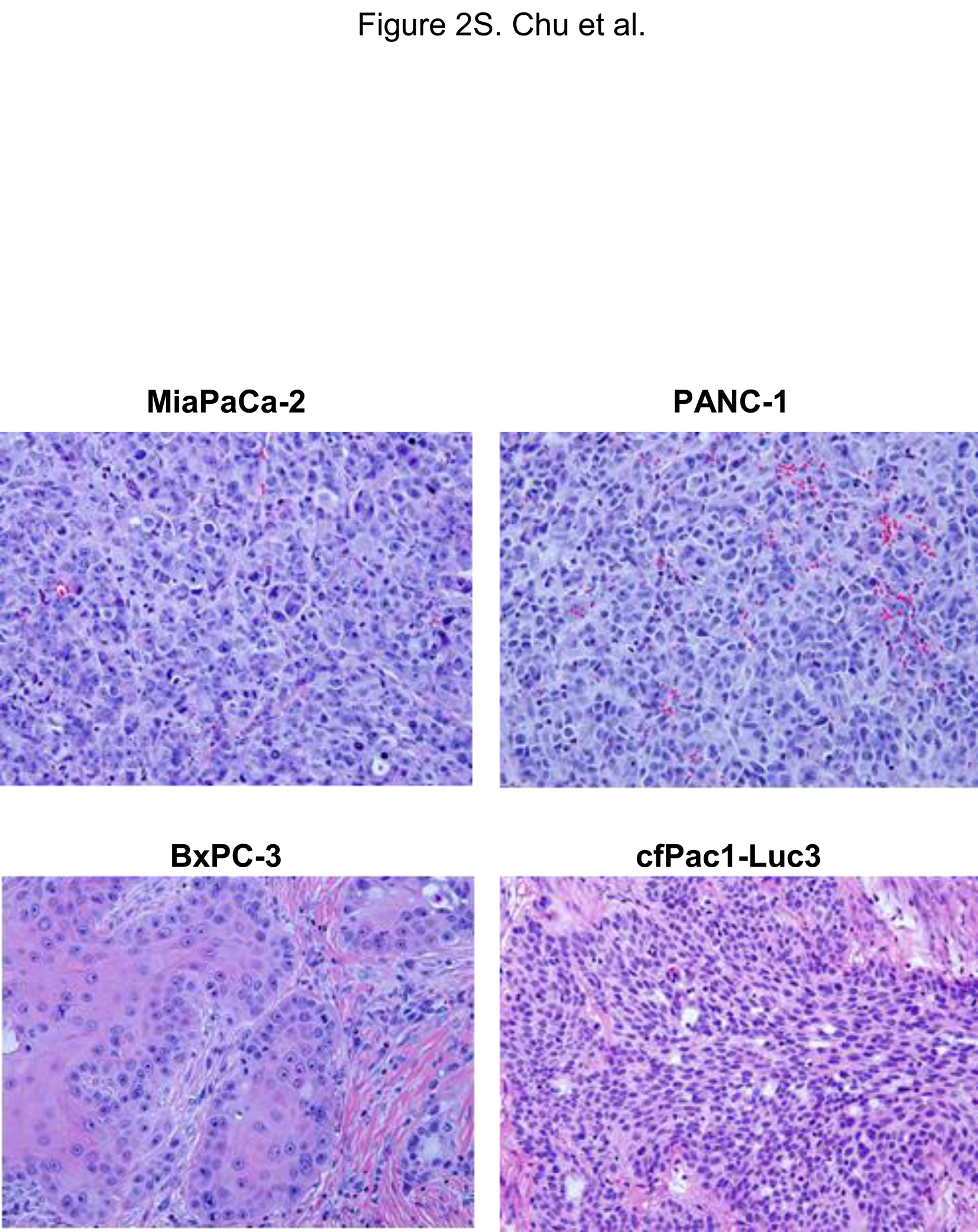

Supplement: Figure S2 — H & E staining of xenografted human pancreatic tumors. (A), (B), (C) and (D) show the xenografts of MiapaCa-2, PANC-1, BxPC-3 and cfPac1-Luc3, respectively, using 400× magnification. (TIF) [file pone.0075507.s002.tif]
