# Supplementary material for: Targeting and Cytotoxicity of SapC-DOPS Nanovesicles in Pancreatic Cancer
Source: PLoS One. 2013 Oct 4;8(10):e75507. doi: 10.1371/journal.pone.0075507 (PMC3790873; doi:10.1371/journal.pone.0075507)
Supplement: Materials S1 — (DOCX) [file pone.0075507.s003.docx]

**Supplementary materials text**

**Orthotopic pancreatic tumor cell injection techniques:**

Female nude mice were anesthetized with isoflurane. A small left abdominal flank incision was made and the spleen exteriorized. Tumor cells (cfPac1-Luc3, 1X10^6^ cells in 30 µl PBS) were injected subcapsularly in a region of the pancreas just beneath the spleen. We used a 30-gauge needle, a 1-ml disposable syringe. A successful sub capsular intrapancreatic injection of tumor cells was identified by the appearance of a fluid bleb without intraperitoneal leakage (Figure 1S). To prevent such leakage, a cotton swab was held for 1 min over the site if injection. Abdominal wound was closed in two layers, peritoneum with vicryl 4-0 and skin closed with the silk 4-0 sutures. For generating orthotopic pancreatic tumor model, adult female mice weighing ~20-25 g (Taconic farms, Germantown, NY) mice were anesthetized with isoflurane. A small left abdominal flank incision was made and the spleen exteriorized.

**Supplement figure legends:**

**Figure 1S: Orthotopic pancreatic tumor cell injection techniques.** (A) A small left abdominal flank incision was made and the spleen exteriorized. (B) A successful subcapsular intrapancreatic injection of tumor cells was identified by the appearance of a fluid bleb without intraperitoneal leakage.

**Figure 2S: H&E staining of xenografted human pancreatic tumors.** (A), (B), (C) and (D) show the xenografts of MiapaCa-2, PANC-1, BxPC-3 and cfPac1-Luc3, respectively, using 400x magnification.
